# Cultural Intelligence and Work–Family Conflict: A Moderated Mediation Model Based on Conservation of Resources Theory

**DOI:** 10.3390/ijerph16132406

**Published:** 2019-07-06

**Authors:** Guohua He, Ran An, Feng Zhang

**Affiliations:** 1 School of Business Administration, South China University of Technology, Guangzhou 510640, China; 2 Desautels Faculty of Management, McGill University, Montreal, Québec H3A 1G5, Canada; 3 School of International Education, South China University of Technology, Guangzhou 510000, China

**Keywords:** cultural intelligence, work–family conflict, Chinese expatriates, Confucius Institute, conservation of resources

## Abstract

This study aims to explore the influence mechanism of cultural intelligence on work–family conflict for Chinese expatriates in cross-cultural non-profit organizations. Drawing on conservation of resources theory, this longitudinal study (six-month time lag) is the first to examine cultural intelligence as an antecedent of work–family conflict. The study also examines the mediating role of work engagement and the moderating role of leader–member exchange (LMX) in the cultural intelligence and work–family conflict relationship. The sample comprises 206 expatriate Chinese language teachers working at 45 Confucius Institutes in the US, Canada, and Russia. Results show that cultural intelligence not only reduces work–family conflict but also promotes expatriates’ work engagement. The higher the work engagement, the higher the work–family conflict experienced by expatriates. LMX moderates not only the positive relationship between work engagement and work–family conflict but also the indirect effect of cultural intelligence on work–family conflict through work engagement. Thus, the indirect effect of cultural intelligence on work–family conflict through work engagement is stronger with low (compared to high) LMX. This study’s findings provide implications for managers of cross-cultural non-profit organizations to better understand and solve expatriates’ work–family conflict problem.

## 1. Introduction

Accelerating internationalization made transnational cooperation in education a typical feature of many Chinese universities. Academic expatriates are, therefore, becoming increasingly popular, progressively raising their importance in human resource management [1]. However, previous work–family conflict research mostly focuses on expatriates in profit-making organizations, such as multinational enterprises (e.g., [2,3,4]). Studies on academic expatriates in cross-cultural non-profit organizations (NPOs) are rare, leaving organizations ill-informed on how to best promote employee well-being [5]. Research on the work–family issues of employees provides valuable practical implications for NPOs to attract and retain high-quality employees, because employees prefer to seek for jobs that are both meaningful and well balanced between work and family obligations [6]. Pitt-Catsouphes et al. [7] argue that since women form the majority of NPO employees, work–family conflict is especially prevalent in the non-profit sector. Compared with enterprises or other profit-making organizations, NPOs provide fewer benefits and supports to employees [8]. Due to resource constraints, it is often difficult for NPOs to meet employees’ needs for balancing work–family relationships. Therefore, understanding the work–family conflict of academic expatriates in cross-cultural educational organizations is vital for enhancing their well-being and enriching the existing literature on international human resource management [9].

With the increasing cross-border mobility of the global labor force and the popularity of dual-earner families, work–family conflict has become a prominent societal concern, attracting growing attention from researchers of various disciplines (e.g., [10,11]). Relevant surveys have found that family concerns are the primary reason for employees to refuse expatriation [12]. Work–family conflict reflects the extent to which work and family roles are mutually incompatible [13]. Family is arguably a very important influencer of expatriation success, which not only challenges expatriates themselves but also changes their work and family life [14]. Employees’ ability to balance work and family relationships will affect whether they can successfully complete international assignments [15]. Most work–family conflict studies explore its outcomes or mediating role, while its antecedent variables are mainly investigated through meta-analyses. For example, Michel et al. [16] showed that job involvement, social support, and personality all influence work–family conflict. Byron [17] classified the antecedent variables of work–family conflict into three categories: Work domain, non-work domain, and demographic variables. Other meta-analyses have identified stress and dispositional variables as work–family conflict antecedents [18,19]. Whereas work–family conflict antecedents have been comprehensively and systematically summarized, few studies have tested work–family conflict as the dependent variable or how a specific variable relates to work–family conflict, especially for expatriates in cross-cultural NPOs. Exploring this under-researched context may provide a new perspective on human resource management and deepen our understanding of work–family conflict.

Differences in the impact of culture on work–family conflict experience make it more complex for expatriates in cross-cultural NPOs compared to those in general multinational enterprises [20]. In particular, where expatriate work involves extensive cross-cultural communication and interaction, cultural intelligence is especially important. Claus et al. [8] argue that NPOs tend to provide fewer benefits and organizational support to expatriates than multinational corporations; consequently, expatriates in NPOs must rely on their own resources and other support mechanisms to meet both work and life challenges. Cultural intelligence, as a personal resource, may in fact be more significant and bring advantages in work and life for expatriates working for cross-cultural NPOs. However, the “black box” of cultural intelligence’s influence on work–family conflict among expatriates needs opening. Cultural intelligence concerns an individual’s ability to function and manage effectively in a cross-cultural context [21]. It transcends the general scope of intelligence, emphasizing ability to recognize, absorb, and reason correctly when handling problems in a cross-cultural environment [22]. Most studies on cultural intelligence adopt a positive organizational behavior perspective, exploring its various positive outcomes, especially cross-cultural adjustment and performance [23,24]. As a relatively new concept only recently attracting scholarly attention, empirical research on cultural intelligence remains limited, especially studies on its “dark side” or its relationship with negative outcome variables [23]. According to conservation of resources (COR) theory, anything that helps to achieve personal goals can be called a resource [25]. Accordingly, cultural intelligence can be considered a valuable resource that helps expatriates to achieve goals in a cross-cultural workplace [26]. Cultural intelligence has already been identified as an important ability enabling expatriates to perform tasks, while work–family conflict has long been a concern for researchers and managers; however, whether cultural intelligence affects expatriates’ attitudes or behaviors in the family domain remains to be studied. Therefore, one purpose of this study is to explore, from the perspective of COR theory, how cultural intelligence affects expatriates’ work–family conflict in cross-cultural NPOs.

Work engagement is a motivational concept, which reflects employees’ voluntary allocation of physical, cognitive, and emotional resources in work and related activities [27]. The positive outcomes of work engagement have been extensively studied, and include reducing stress and depression [28], improving sleep quality [29], and promoting employee innovation and organizational performance [30]. Although a few studies suggest the possible dark side of work engagement (e.g., [31,32,33]), empirical research is rare. Shimazu et al.’s [34] findings suggest that overly high work engagement can detrimentally impact employees’ psychological distress and job performance. Similarly, Sonnentag [33] claimed that work engagement may be curvilinearly related to positive outcomes, such that excessive work engagement may be harmful. The antecedents of work engagement usually involve employees’ perception of working conditions, which mainly includes two categories: Job demands and resources [35]. Several authors have suggested that engagement results from high levels of resources (e.g., [36,37]). According to Halbesleben et al. [25], resources include anything that individuals perceive helpful for attaining working goals, such as energy, cultural skills, intrinsic motivation, leader support, and work control. Cultural intelligence is a valuable personal resource that enables employees to interact and cooperate effectively with people from different cultures. Employees with high cultural intelligence could better adapt to cross-cultural working conditions and develop more positive emotions at work. Therefore, it is reasonable to infer that, as a resource, cultural intelligence promotes employees’ work engagement, but too much engagement may negatively impact the non-work domain as each individual possesses limited resources. High work engagement has been found to significantly and positively correlate with work–family conflict (e.g., [2,38]), but limited research on the antecedent variables of work engagement prevents deeper understanding of this relationship. As a desirable outcome intensively pursed by organizations, the above studies indicate that organizations face the dilemma of motivating employees to increase their work engagement while avoiding its dark side. Discovering how managers or organizations can overcome this dilemma, focusing especially on the role of contextual characteristics as a boundary condition for the work engagement and work–family conflict relationship, is of great significance to employee well-being and organizational performance [39].

Since organizational behavior can usually be explained through the interaction of individual characteristics and contextual variables [40], research on how their interaction affects work–family conflict is needed to better understand expatriates’ work–family conflict. In this paper, leader–member exchange (LMX) was proposed as a contextual variable, which refers to the different quality of exchange relationships that developed between leaders and their subordinates [41]. Accordingly, this paper explores the influence mechanism in the cultural intelligence and work–family conflict relationship, and examines how the contextual variable of LMX functions as a boundary condition for this relationship in cross-cultural NPOs.

This study’s contribution to the work–family conflict literature is threefold. First, it provides the first validation of cultural intelligence as a work–family conflict antecedent for academic expatriates working in cross-cultural NPOs. In theoretical terms, it gives HRM researchers and HR managers a better understanding of the importance of cultural intelligence in reducing work–family conflict. Second, this study verifies work engagement as a mediating variable in the relationship between cultural intelligence and work–family conflict. This investigation explains why cultural intelligence promotes work–family balance and facilitates understanding of the process through which cultural intelligence impacts on work–family conflict. Third, this study reveals that LMX moderates the positive relationship between work engagement and work–family conflict. It shows that although cultural intelligence positively influences employees’ work engagement, the importance of high-quality LMX in reducing the negative effect of excessive work engagement should not be neglected.

## 2. Theoretical Background and Hypotheses

### 2.1. Conservation of Resources Theory

COR theory proposes that people acquire, invest, and protect resources that they value to cope with depression and stress. Resources are loosely defined as any competencies, objects, personal characteristics, energies, or conditions that people value [42]. Criticisms of this vague definition have appeared in many publications (e.g., [43]). According to Halbesleben et al. [25], resources have been defined as anything perceived by employees that helps them attain goals. This recently proposed goal-based definition enables better understanding of the characteristics of resources and how they promote goal attainment.

Employees are motivated to preserve their resources and invest them to gain additional resources; the loss or gain of resources depends on possessing other surplus resources [44]. Since the 1990s, COR theory has been frequently used to study the work–family relationship. It holds that when workers invest too many of their limited resources in one role, they have less resources available for other roles. Workers will try to protect or recover their resources by investing more resources (e.g., time, energy, money) in the role threatened by resource loss. For employees, time and energy are finite resources, so it can be difficult to manage both family and work roles due to their conflicting time and energy demands or incompatible required behaviors [13]. Kossek et al. [45] argued that the depletion of resources in work can lead to resource scarcity and difficulty in meeting family domain demands, thus raising work–family conflict. Therefore, it can be inferred that employees with more resources experience lower work–family conflict.

There are two primary tenets of COR theory: Resource acquisition and resource conservation [2]. Resource acquisition suggests that individuals actively interact with their surrounding environment to increase their resource reserves [46]; resource conservation indicates that, to avoid resource loss or potential future threats, individuals are motivated to withdraw from situations or behaviors considered to threaten their resources. COR theory also predicts that employees try to maximize resource accumulation through strategic investment of resources [47], such as exhibiting high job engagement or work performance with the aim of promotion or rewards. Thus, resources obtained from the workplace are often reinvested in work-related behaviors [2,47]. Importantly, Hobfoll [47] proposed two corollaries of the resource investment tenet of COR theory. The first indicates that individuals with resource surpluses are less vulnerable to resource losses and can better invest existing resources; the second holds that lacking resources makes investment more difficult and increases vulnerability to resource losses.

Since Hobfoll [42] first proposed COR theory, researchers have described and studied various resources. Recent studies have treated cognitive cultural intelligence as a psychological resource that helps promote expatriates’ career engagement and life satisfaction [48]. Grandey and Cropanzano [49] contended that COR theory largely explains individual differences in the work–family conflict process. Individual differences can affect responses to stress or resource loss. For example, individuals with high cultural intelligence can more effectively cope with cross-cultural challenges; they are also less susceptible to resource loss as they know they can cope therewith [48]. Drawing from COR theory, this study holds that expatriates with high cultural intelligence are motivated to invest resources at work to gain more resources. By developing a moderated mediation model, we further explore the underlying mechanism of cultural intelligence’s influence on work–family conflict among Chinese academic expatriates.

### 2.2. Cultural Intelligence and Work–Family Conflict

Since its introduction by Earley [50], the cultural intelligence concept has become a popular topic of scholarly conversation and multidisciplinary research [24]. As a kind of cross-cultural competence, cultural intelligence can be predicted by personality traits and explains why individuals are more effective in a certain cultural context [51]. Van der Laken et al. [26] proposed that cultural intelligence helps expatriates achieve goals in a new cultural environment or workplace. Cultural intelligence is typically regarded as a multi-dimensional construct, distinguished from general intelligence [24]. Earley and Ang [21] proposed four constituent aspects of cultural intelligence: Metacognitive, cognitive, motivational, and behavioral. Metacognitive cultural intelligence refers to the psychological process through which people acquire and understand cultural knowledge; cognitive cultural intelligence emphasizes the structure and content of cultural knowledge; motivational cultural intelligence reflects the extent to which an individual values cross-cultural interaction and active use of cultural intelligence; and behavioral cultural intelligence concerns the ability to use appropriate language and non-language behaviors in cross-cultural interactions. Together, these aspects constitute an individual’s overall ability to effectively function in a multicultural environment [21]. Based on them, Ang et al. [22] have developed a cultural intelligence measurement, which has been widely used in subsequent studies.

Thomas et al. [52] emphasize the integrity of cultural intelligence, which they define as a higher order concept generated by the interaction of cultural knowledge, cross-cultural skills, and cultural metacognition. It reflects an individual’s ability to interact effectively in different cultural contexts or with people from different cultures. Thomas et al. [53] also developed a short-form measure of cultural intelligence (SFCQ) containing 10 items, claimed to accurately reflect the complexity of cultural intelligence and effectively predict individual cross-cultural effectiveness. Indeed, SFCQ is highly suitable for studying the integrity of cultural intelligence, being an integral construct whose dimensions cannot be separated from one another [24]. Most studies have treated cultural intelligence as an integral construct, emphasizing the value of studying it as a whole [54]. Consistent with previous research (e.g., [51,55]), this study treats cultural intelligence as an integral concept and an individual resource.

Work–family conflict is a form of inter-role conflict caused by incompatible work and family demands. Essentially, participating in the work (family) role is made more difficult by participating in the family (work) role [13]. Work–family conflict comprises two highly related paths with opposite directions: Work interference with family (WIF) and family interference with work (FIW) [56]. Greenhaus and Beutell [13] proposed three major types of work–family conflict: Time-based, stress-based, and behavior-based. Time-based conflict refers to how time spent on one role affects one’s duty to fulfill another role. Stress-based conflict refers to the possible transfer of stress from one role to another, thus hindering the fulfillment of the latter’s obligations. Behavior-based conflict refers to the inconsistency between behaviors needed to fulfill two roles’ distinct requirements [18]. Since this paper focuses on work-related variables, only the WIF direction is considered. WIF is “a form of inter-role conflict in which the general demands of, time devoted to, and strain created by the job interfere with performing family-related responsibilities” [57]. Existing research emphasizes the difference between WIF and FIW and holds that work-related variables are more closely related to the former [17]. Moreover, LMX and work engagement have been found to be more closely related to WIF rather than FIW [58,59]. Therefore, in this paper, work–family conflict only refers to the conflict caused by WIF.

According to COR theory, employees with more resources are less likely to lose them and more likely to attain additional resources [60]. Therefore, employees with more resources (like energy, time, etc.) should be less likely to experience work–family conflict. COR theory emphasizes the decisive role of culture as a kind of resource [61]. Everything that individuals strive to acquire or wish to retain can be considered resources [62]. Cultural intelligence can, thus, be regarded as a valuable resource that helps employees cope with various challenges in a cross-cultural environment. Compared to low cultural intelligence employees, employees with high cultural intelligence have a greater resource surplus and are more capable of gaining additional resources through resource investment or allocation; they can adapt to different cultures and workplaces more quickly with the resources they possess. Therefore, high cultural intelligence employees have more time, energy, and other resources to balance work–family relationships and reduce the occurrence of work–family conflict. This view is consistent with those expressed in other studies. For example, Rao [63] indicated that competence, intelligence, and education level may affect work–family balance. Accordingly, cultural intelligence could help employees achieve work–family balance and promote the sustainable development of human beings. Yang et al. [4] showed an inverse relationship between employees’ job-related resources and the work–family conflicts they experienced. Brislin et al. [64] argued that employees with high cognitive cultural intelligence can better understand the similarities and differences between cultures and more effectively handle interpersonal relationships in cross-cultural environments, thus enhancing their satisfaction with family life. All these studies indicate a negative relationship between cultural intelligence and work–family conflict. Therefore, the following hypothesis is proposed.

**Hypothesis** **1:** *Cultural intelligence is negatively related to employees’ work–family conflict*.

### 2.3. Mediating Role of Work Engagement

Work engagement has become a popular topic for organizations and researchers, especially as it usually brings desirable outcomes for different types of organizations and individuals [65]. Many studies have explored the antecedents and outcome variables of work engagement (e.g., [29,30]). Although most studies focus on the relationship between work engagement and positive outcomes, Sonnentag [33] advocated the importance of exploring the dark side of work engagement, thereby deepening understanding of work engagement and its relations with individual or organizational factors. Recent research revealed a U-shaped relationship between work engagement and psychological stress, and an inverted U-shaped relationship between work engagement and performance [34], suggesting the possible negative impact of overly high work engagement. However, few empirical studies have explored work engagement’s dark side [34].

Schaufeli et al.’s [66] definition of work engagement—“a positive, fulfilling, work-related state of mind that is characterized by vigor, dedication, and absorption”—is the most widely recognized. Vigor refers to energy and resilience in work; dedication means one’s deep involvement in work and in experiencing associated feelings of significance, enthusiasm, and challenge; and absorption refers to the state of being totally absorbed and actively engaged in work. Although work engagement is a multi-dimensional concept, this study examines it as an integral construct, consistent with most of the literature (e.g., [65,66]).

This study predicts that work engagement plays a mediating role in the cultural intelligence and work–family conflict relationship. First, we propose that cultural intelligence can promote expatriate employees’ work engagement in a cross-cultural work context. According to COR theory [42], employees with more resources are likelier to make investments to obtain more resources. Research shows that work engagement results from individuals possessing high levels of resources [37]. As a resource owned by employees, cultural intelligence should motivate them to further invest resources in their work. Knight et al. [67] proposed that work engagement is driven by work or personal resources, which motivate employees to be more engaged in work or enhance their well-being. As a valuable resource, cultural intelligence helps expatriates to alleviate the anxiety and pressure of cultural differences, facilitating their quicker adaptation to other cultures and more effective interaction in cross-cultural workplaces [21,64]. In turn, these outcomes contribute to employees’ work engagement, as other recent studies have contended. For example, Le et al. [48] argued that cognitive cultural intelligence, as a psychological resource, can help expatriates feel more comfortable and confident in a multicultural workplace, thus promoting their work engagement. Boštjančič et al. [68] proposed that work and personal resources contribute to employee work engagement and help employees obtain more resources. Furthermore, Hakanen et al. [69] asserted that teachers are motivated to invest resources and enhance their work engagement if they possess resources that help them meet job demands. Other studies have also revealed a positive relationship between employees’ job-related resources and their work engagement (e.g., [2,70]). Therefore, as a resource possessed by workers, cultural intelligence is expected to effectively promote employee work engagement in a multicultural work environment.

Second, although work engagement is a positive and desirable behavior for both employees and managers [2], studies have indicated that continuous work engagement is very difficult because employees’ available resources and abilities are limited; devoting resources to the work domain reduces investment in the family domain [71]. Therefore, work engagement may lead to work–family conflict. According to COR theory [42], if employees expend too many resources (e.g., time, energy) in one role, resource investment in another role will reduce, inhibiting their ability to meet the latter role’s demands. Employees face the problem of work–family conflict if they lack sufficient resources to cope with demands from both the work and family domains. Therefore, when employees’ work engagement is high, they invest a large amount of time, energy, and other resources into their work, leaving fewer resources available for devoting care and attention toward their families, thus raising the likelihood of work–family conflict [42]. Torp et al. [38] showed that high job demands are significantly and positively correlated with work–family conflict among university scholars, while Halbesleben et al. [2] proposed that high work engagement leads to more WIF. These findings are reinforced by other studies (e.g., [31,72]). Based on the above discussion, the following hypothesis is proposed:

**Hypothesis** **2:** *Work engagement mediates the negative effect of cultural intelligence on work–family conflict*.

### 2.4. Moderating Effects of LMX

LMX concerns the quality of the exchange relationship between leaders and subordinates based on trust, respect, and obligation [73]. LMX theory holds that the exchange relationships that develop between leaders and their subordinates differ in quality. High-quality LMX is based on mutual trust, respect, and common obligations between leaders and subordinates [74]. It helps employees to attain more resources (such as social support), protects them from threats or stress, and is of great value in alleviating work–family conflict among expatriates [75]. Work–family conflict research emphasizes leaders’ influence on employees’ work–family balance, especially the role of social support from leaders [3]. The latest meta-analysis of work–family conflict showed that leader support or other psychological and material support in the workplace can reduce employees’ work–family conflict, with work support the most important factor for reducing work–family conflict in collectivist countries [76]. Since the relationship between teachers’ individual characteristics and behavior is likely affected by their work environment [41], this study proposes that employees with high-quality LMX have lower work engagement-driven work–family conflict than those with low-quality LMX.

According to COR theory, resources available from organizations or individuals can effectively alleviate employees’ pressure when they perform multiple roles. As a valuable resource possessed by workers, high-quality LMX helps to compensate for resources lost (e.g., emotional exhaustion) through work–family conflict driven by excessive work engagement. In a high-quality LMX relationship, leaders understand employees’ difficulties and needs, and are willing to provide the resources they need. Leaders are important sources of workplace social support for employees [77], which helps them devote more time, energy, and other resources to the family domain and meet family role expectations. Therefore, work support from leaders reduces employees’ work–family conflict [78]. Furthermore, in a high-quality LMX relationship, leaders are more likely to voluntary help employees solve their work–family conflict problems, such as by formulating supportive working policies, offering flexible shifts, and allowing employees to choose workplaces and working hours [75].

By contrast, low-quality LMX relationships are based on economic exchange, with employees performing their duties and receiving corresponding payment according to their employment contracts [73]. Employees with low-quality LMX are more vulnerable to resource loss and invest inadequate resources in work when facing resource depletion caused by role conflicts. Such employees are considered “out-group” members by leaders, who hardly recognize their contributions; they also feel unable to rely on their leaders to provide necessary resources [74]. Thus, low-quality LMX is characterized by low levels of trust, mutual respect, and obligations between leaders and their subordinates, with the latter struggling to obtain support from leaders or the organization to balance their work and family life. In particular, when employees engage in work excessively, limited resources such as time and energy are disproportionately invested in the work domain, leaving less available for the family domain. In such circumstances, inability to balance resources between the work and family domains, or if resource consumption in the family domain cannot be supplemented by other resources, it leads to work–family conflict. Therefore, employees’ work engagement is likely to induce higher work–family conflict in low-quality (compared to high-quality) LMX relationships.

The moderating effect of LMX on the work engagement and work–family conflict relationship can also be explained by trait activation theory [79], which posits that both personal and contextual characteristics could affect behavior. This theory explains behavior as responses to “trait related cues” in a given situation. Translated to our study, a situation can provide a high or low number of cues for the work–family conflict of expatriates with high work engagement. LMX is a trait-related cue in the workplace and influences the work–family conflict of engaged expatriates [41]. Based on prior findings on “situation strength” [80], this study proposes that the above-mentioned trait-related cues could weaken the relationship between work engagement (trait) and work–family conflict (behavior).

Behaviors in weak situations largely depend on personal traits, rather than situational characteristics; the opposite is true for strong situations [81]. Hochwarter et al. [82] found that when employees perceive low organizational support, work behavior can be predicted by individual differences in social skills. Similarly, Runhaar et al.’s [41] research suggests that LMX weakens the relationship between personal trait and behavior. Accordingly, this study suggests that the more trait-related cues (i.e., LMX) an expatriate perceives in their work, the weaker the relationship between their work engagement (personal trait) and work–family conflict (behaviors). In other words, the work situation is expected to have a compensatory effect by strongly influencing relatively low-engaged expatriates. Based on the above argument, the following hypothesis is proposed:

**Hypothesis** **3:** *LMX negatively moderates the positive relationship between work engagement and work–family conflict: The positive work engagement and work–family conflict relationship is weaker in high-quality LMX relationships than in low-quality LMX relationships*.

Based on H1 (Hypothesis) to H3, this study further proposes a moderated mediation model. Employees who exhibit work engagement are more likely to be appreciated by their leaders and develop high-quality LMX with them. Although engaged employees are more likely to face role conflicts, and thus work–family conflict, high-quality LMX can provide employees with psychological security and help them obtain more support from leaders or the organization to balance work and family relations, thus reducing the likelihood of work–family conflict. Therefore, high-quality LMX weakens both the positive relationship between work engagement and work family conflict and the indirect effect of cultural intelligence on work–family conflict through work engagement. Additionally, high cultural intelligence employees are more likely to develop high-quality LMX relationships with leaders [55], and the combined effect of these two factors can significantly reduce employees’ work–family conflict. By contrast, for employees in low-quality LMX relationships, the effect of cultural intelligence on work–family conflict depends more on the mediating role of work engagement. From the above discussion, it can be further inferred that cultural intelligence’s indirect effect on work–family conflict through work engagement is weaker for employees with high-quality LMX than for those with low-quality LMX. Thus, the following hypothesis is proposed.

**Hypothesis** **4:** *The indirect effect of cultural intelligence on work–family conflict through work engagement is negatively moderated by LMX: This indirect relationship is stronger for employees with low-quality LMX than for those with high-quality LMX*.

**Figure 1:**
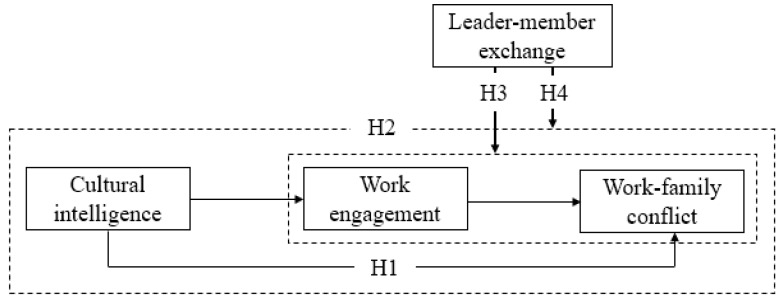
Theoretical model.

Based on the above arguments, the following theoretical model is proposed (Figure 1).

## 3. Method

### 3.1. Participants and Procedures

This study surveyed expatriate Chinese language teachers in Confucius Institutes in the US, Canada, and Russia. Confucius Institute is a cross-cultural NPO dedicated to promoting Chinese language teaching and Chinese culture worldwide. As a Sino-foreign cooperative organization, each Confucius Institute includes both Chinese and foreign employees, entailing extensive cross-cultural communication and interaction both within and outside the organization. Every year, many Chinese language teachers and volunteers are expatriated from China to Confucius Institutes worldwide to teach Chinese as a foreign language or organize Chinese cultural events abroad. These features provide a good context and sound bases for studying the cultural intelligence of expatriate Chinese language teachers. By the end of 2018, 548 Confucius Institutes and 1193 Confucius Classrooms have been established in 154 countries worldwide, and a total of 105,000 expatriate Chinese language teachers were sent to them [83].

The researchers worked as a visiting scholar at a renowned Canadian university from 2017 to 2019. This experience enabled the researchers to conduct field research in 13 Confucius Institutes in the US, Canada, and Russia, thereby building a wide acquaintance network of expatriate Chinese language teachers in these three countries. By number of Confucius Institutes, these three countries rank in the global top 10, with thousands of Chinese teachers expatriated to these three countries every year. The US has the largest number of expatriate Chinese language teachers in the world, which is an overwhelming advantage when compared with the numbers in other countries. In addition, these three countries respectively represent North America and Europe, which are the most concentrated areas for expatriate Chinese language teachers. Samples from these countries are considered to be well representative.

This study was reviewed and approved by the affiliated university’s Research Ethics Board before data collection. Data were collected in time-lagged stages. We used the above mentioned acquaintance social networks to recruit survey participants through snowball sampling. Potential participants were contacted through groups in WeChat (a very popular messaging app among Chinese). Every expatriate Chinese language teacher in the three studied countries belongs to at least one WeChat group. After explaining the research purpose and assuring that response confidentiality would be protected, we sent a link to our online questionnaires, which was generated by a professional survey company in China (Wenjuanxing).

Our research design included two measurement times with a six-month interval. This mainly served to minimize common method variance concerns, and a six-month lag is considered appropriate in work–family conflict research [84,85]. At Time 1, cultural intelligence, LMX, and demographic variables were measured, with 401 expatriate Chinese language teachers participating in the first-wave survey. Approximately six months later, work engagement and work–family conflict were measured in the second-wave survey at Time 2. The 401 first-wave survey participants were invited to complete the second-wave survey using the email address or WeChat number they had provided. In total, 206 Chinese teachers (response rate = 51.37%) from 45 Confucius Institutes in the US, Canada, and Russia participated in the second-wave survey. In each wave, IP address restriction prevented any individual submitting more than one questionnaire. Participants who successfully submitted their online questionnaire in each wave were sent a small monetary reward via WeChat.

Regarding sample composition, 69.4% were female and 30.6% were male. Further, the US accounted for 60.7%, Canada 20.9%, and Russia 18.4% of all respondents. Regarding age, 38.8% of respondents were below 25 years; 9.7% were 26–30; 3.9% were 31–35; 18.4% were 36–40; 23.3% were 41–45; and 5.8% were 46 years and above. Regarding marital status and education level, 44.2% were unmarried, 52.4% were married, and 3.4% were divorced, while 6.3% were undergraduates, 62.1% were master’s students or graduates, and 31.6% were Ph.D. students or graduates. In terms of role, 3.9% were Chinese directors in a Confucius Institute, 20.4% were Chinese teachers expatriated by Hanban, 18.9% were immigrant Chinese teachers, 51% were volunteers, and 5.8% were Chinese administrative assistants.

### 3.2. Measures

All the measures used in this study were scales developed and validated in previous studies. We conducted rigorous two-way translation of the original English measures to ensure translation quality. In addition, to ensure that all the items are suitable and applicable to the research context, some minor modifications were made following suggestions from three professors in a relevant research field and 15 expatriate Chinese language teachers. All measures were scored using a 5-point Likert scale, ranging from 1 (“strongly disagree”) to 5 (“strongly agree”).

#### 3.2.1. Cultural Intelligence

This construct was measured using the SFCQ developed by Thomas et al. [53]. Cross-cultural validation of this scale showed that it had good content and structural validity. This scale contains 10 items: E.g., “I know the ways in which cultures around the world are different.” The construct’s Cronbach’s alpha was 0.91.

#### 3.2.2. Work–Family Conflict

To measure work–family conflict, a 5-item scale developed by Netemeyer et al. [57] was employed. A sample item is “My job produces strain that makes it difficult to fulfill family duties.” This construct’s Cronbach’s alpha was 0.83.

#### 3.2.3. Work Engagement

To measure work engagement, the 9-item Utrecht Work Engagement Scale was used: First developed by Schaufeli et al. [66], it has become the most widely used scale for measuring work engagement. A sample item is “I am proud of the work that I do.” The construct’s Cronbach’s alpha was 0.85.

#### 3.2.4. LMX

This was assessed using Graen and UhI-Bien’s [73] 7-item scale, in which high scores indicate high-quality LMX. A sample item is “My leader can well recognize my potential.” This construct’s Cronbach’s alpha was 0.94.

#### 3.2.5. Control Variables

Some studies have indicated that demographic variables have little effect on estimations of work–family conflict parameters (e.g., [17]). Spector and Bannick [86] argued that demographic variables should not be controlled if they are theoretically unimportant or have little effect on outcome variables. However, we found gender, age, education level, country, and service duration at a Confucius Institute to moderately correlate with work–family conflict (see descriptive statistics reported in Table 1 below), confirming prior findings that these variables influence work–family conflict (e.g., [4]). Therefore, these variables are included as control variables in this study.

## 4. Results

### 4.1. Common Method Variance Tests

Data collected in this study were self-reported, which could introduce common method variance (CMV). Several methods were adopted to minimize this problem, such as reverse coding of scale items, anonymous answering, and counterbalancing question order. Meanwhile, Harman’s single-factor analysis was employed to test for CMV. All the study’s variables were loaded into an exploratory factor analysis using the SPSS 22.0 software (IBM, Armonk, NY, USA). The factor analysis without rotation revealed nine factors with an eigenvalue exceeding 1, while the cumulative variation explained by the first factor was 14.14% (i.e., lower than the critical value of 50%). This indicated that CMV was within an acceptable range. Following the method suggested by Podsakoff et al. [84], CMV was further tested by controlling for the effects of an unmeasured latent factor. All the items were loaded on their corresponding constructs, as well as the unmeasured latent factor, and then the fitness index of the five-factor model was compared with that of the four-factor model. Specifically the goodness of fit statistics was evaluated with indexes of Comparative Fit Index (CFI), Tucker-Lewis Index (TLI), Incremental Fit Index (IFI), and Root Mean Square Error of Approximation (RMSEA). Although the five-factor model with an unmeasured latent factor (*x*^2^ = 728.358, *df* = 298, *x*^2^/*df* = 2.444, CFI = 0.921, TLI = 0.909, IFI = 0.922, RMSEA = 0.072) has a better model fit than the four-factor model, the improvement is small (see Table 2), again indicating that there is no serious CMV problem in this study.

### 4.2. Confirmatory Factor Analysis

Amos 20.0 was used to examine the discriminant validity of cultural intelligence, work–family conflict, work engagement, and LMX through confirmatory factor analysis (CFA). First, a baseline model containing all four variables was built. Its fitness was then compared with three alternative models. The results show that the baseline model has the best fitness degree (*x*^2^ = 876.528, *df* = 318, *x*^2^/*df* = 2.756, CFI = 0.914, TLI = 0.905, IFI = 0.914, RMSEA = 0.078), indicating that the four constructs have good discriminant validity.

### 4.3. Descriptive Statistical Analysis

The mean, standard deviation, and correlation coefficient of each study variable are reported in Table 1. Cultural intelligence was significantly correlated with work engagement (*r* = 0.337, *p* < 0.01) and work–family conflict (*r* = −0.450, *p* < 0.01), while work engagement was positively correlated with work–family conflict (*r* = 0.295, *p* < 0.01). These results provided preliminary support for the study’s hypotheses.

Table 3 presents the results of the hierarchical regression models used to test the study’s hypotheses. To test H1 (predicting a negative relationship between cultural intelligence and work–family conflict), work–family conflict was entered as the dependent variable and the control variables as independent variables in Model 1. The results show that gender and tenure were significantly associated with work–family conflict, whereas age and education level did not affect work–family conflict. Based on Model 1, cultural intelligence was then entered as an independent variable (Model 2). The results suggest that cultural intelligence was negatively and significantly related to work–family conflict (β = −0.560, *p* < 0.001), with 41% of total variance explained by cultural intelligence. Thus, H1 is supported.

To test H2 (predicting that the cultural intelligence and work–family conflict relationship is mediated by work engagement), the procedures suggested by Baron and Kenny [87] were followed. The first step was to test the significant effect of the independent variable (cultural intelligence) on the dependent variable (work–family conflict). The result for H1 confirms that these variables were significantly related. The second step was to test the significant effect of the independent variable (cultural intelligence) on the mediating variable (work engagement). Work engagement was entered as the dependent variable, followed by the control variables (Model 7) and cultural intelligence (Model 8) successively as independent variables. The results of Model 8 show that cultural intelligence has a significantly positive relationship with work engagement (β = 0.488, *p* < 0.001). The third step was to examine the significant effect of the mediator (work engagement) on the dependent variable (work–family conflict), while controlling for the effect of the independent variable (cultural intelligence). Work–family conflict was entered as the dependent variable, followed by the control variables (Model 1) and work engagement (Model 3) successively as independent variables. The results of Model 3 show that work engagement was positively and significantly related to work–family conflict (β = 0.506, *p* < 0.001). However, when both cultural intelligence and work engagement were entered into a regression analysis (Model 4), the results suggest that cultural intelligence was still negatively and significantly related to work–family conflict (β = −0.434, *p* < 0.05), indicating that work engagement partially mediated the relationship between cultural intelligence and work–family conflict.

To further verify work engagement’s mediation effect, PROCESS was used to compute the mediator’s bias-corrected confidence interval. A 5000 iteration Bootstrap resampling was performed, with confidence intervals set at 95%. Cultural intelligence’s indirect effect on work–family conflict through work engagement was −0.273, with the 95% bias-corrected confidence intervals being [−0.325, −0.119]. The bias-corrected confidence intervals do not contain zero, which suggests the significance of the indirect effect. Thus, H2 is supported.

**Figure 2:**
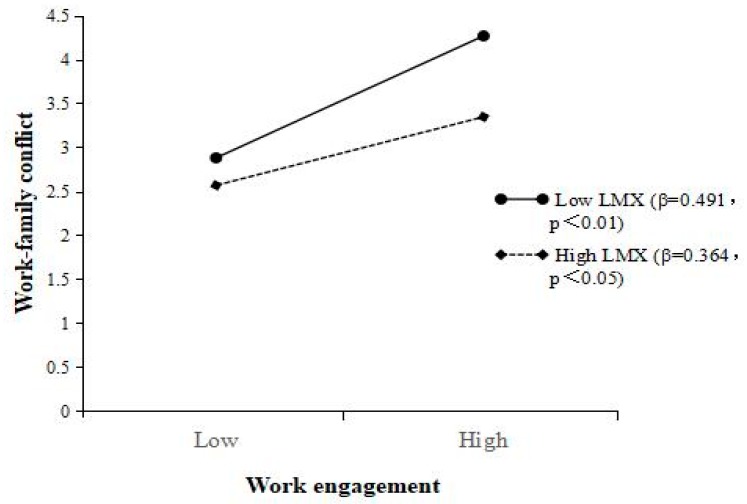
Interactive effects of work engagement and LMX on work–family conflict.

To test H3 (predicting that LMX negatively moderates the positive relationship between work engagement and work–family conflict), moderated regression analysis was employed. The independent (work engagement) and moderating (LMX) variables were first mean-centered to reduce multicollinearity before generating their interaction effects. Model 6 in Table 3 shows that the interaction between work engagement and LMX was negatively related to work–family conflict (β = –0.152, *p* < 0.01). To better understand the moderating effect, we followed Aiken and West’s [88] method to conduct a simple slope analysis. Slopes were computed by adding/subtracting one standard deviation from the mean of LMX, then plotting the interaction patterns. As Figure 2 shows, work engagement was more positively correlated with work–family conflict when LMX was low (β = 0.491, *p* < 0.01) than when LMX was high (β = 0.364, *p* < 0.05). Therefore, H3 is supported.

The moderated mediation effect (H4) was tested following the moderated path analysis suggested by Edwards and Lambert [89]. Table 4 shows that the indirect effect of cultural intelligence on work–family conflict through work engagement varied significantly according to the different levels of LMX (*△*β = −0.211, *p* < 0.05). Specifically, the indirect effect of cultural intelligence on work–family conflict through work engagement was stronger when LMX was low (β = 0.367, *p* < 0.01) than when it was high (β = 0.156, *p* < 0.01). Thus, H4 is supported.

Furthermore, Table 4 shows the significant moderating effect of LMX in the second stage (the effect of work engagement on work–family conflict, *△*β = −0.304, *p* < 0.05). The effect of work engagement on work–family conflict was weaker when LMX was high (β = 0.287, *p* < 0.01) than when it was low (β = 0.591, *p* < 0.01), which indicates that LMX attenuated the positive effect between work engagement and work–family conflict. This further supports H3. These results also suggest that LMX did not moderate the first stage (the effect of cultural intelligence on work engagement, *△*β = –0.081, n.s.) or the direct effect of cultural intelligence on work–family conflict (*△*β = 0.014, n.s.). Therefore, the above analysis further validates LMX’s moderating effect in the second stage (H3) and the moderated mediation effect through work engagement (H4).

## 5. Discussion

Based on COR theory, a moderated mediation model was developed to explore the influence mechanism of cultural intelligence on work–family conflict among Chinese expatriates. The findings suggest that expatriates’ cultural intelligence is helpful in minimizing their work–family conflict and that work engagement mediates this relationship. LMX was found to negatively moderate the work engagement and work–family conflict relationship, as well as the indirect effect of cultural intelligence on work–family conflict through work engagement.

### 5.1. Theoretical Implications

First, this is the first study to find a significant negative relationship between cultural intelligence and work–family conflict for expatriates in a cross-cultural NPO, thus extending research on cultural intelligence and work–family conflict. Previous studies have mostly examined the positive impact of cultural intelligence on organizational behavior, with cross-cultural adjustment and work performance identified as the two most studied outcomes of cultural intelligence ([23,24]. However, few studies have examined cultural intelligence’s relationship with negative outcomes (e.g., work–family conflict). Despite work–family conflict being among the most prevalent challenges for expatriates, the literature has long neglected whether cultural intelligence affects expatriates’ attitudes and behaviors in the family domain. Drawing on COR theory, this study defines cultural intelligence as a valuable resource that helps to reduce expatriates’ work–family conflict. It further enriches research on cultural intelligence’s outcome variables by examining the negative correlation between cultural intelligence and work–family conflict, thus highlights the importance of cultural intelligence to expatriates, which is lacking in existing research [24]. This study’s validation of the cultural intelligence and work–family conflict relationship is also consistent with Vieira et al. [90], who found that work domain resources are a source of individual motivation and commitment that can effectively promote work–family balance and reduce work–family conflict. In applying cultural intelligence to study expatriates in a cross-cultural NPO, our paper answers Ott and Michailova’s [24] call to strengthen the application of cultural intelligence in groups other than business expatriates, and to examine how it affects expatriates’ family relationships. Therefore, this study fills a gap in current research and further enriches and expands the cultural intelligence literature.

Second, this is also the first study to reveal the influence mechanism of cultural intelligence on work–family conflict among expatriates. It verifies the mediating role of work engagement in the cultural intelligence and work–family conflict relationship, and enriches studies on work engagement’s dark side. Most previous studies have focused on its “bright side,” showing that work engagement helps employees attain more resources, bringing several positive outcomes and a potential gain spiral [91,92]. Although the possible dark side of work engagement has recently been recognized, empirical studies on this topic are scarce [34]. The study’s results show that, although cultural intelligence contributes to expatriates’ work engagement, this does not always result in resource gain or positive outcomes. Excessive resource investment at work indicates inadequate resource investment in the family domain and potential conflicts between these two fields. Therefore, contradicting the positive perspective of COR theory on resources [42,61], this study indicates that possessing resources is not necessarily positive and will not always bring positive outcomes. This study enhances understanding of the role of resources in COR theory and suggests the need for appropriately redefining resources in the future; this is consistent with recent studies that attempt to refine COR theory (e.g., [25,93]). Moreover, the findings confirm COR theory’s dynamic nature, as personal resources show different trajectories over time. Although work engagement usually brings resource attainment and many positive outcomes for individuals, this gaining process fluctuates [25]. This study identifies work–family conflict as a potential factor in an individual shifting from resource gaining to resource depletion.

Besides confirming that the negative relationship between work engagement and work–family conflict is found among Chinese expatriates in a cross-cultural NPOs (e.g., [2,31,38]), this study contributes further by examining the antecedent variables of work engagement, thereby enhancing understanding of the relationship between cultural intelligence, work engagement, and work–family conflict. Furthermore, while previous studies have reported that work engagement is an outcome variable of work–family conflict (e.g., [94,95]), this study’s results show that it can also be an antecedent variable of work–family conflict. This finding corresponds with Amstad et al.’s [96] view that a specific variable could be both an antecedent and outcome variable of work–family conflict; examining this feedback loop can deepen understanding of the work–family process. Therefore, this study also enriches the work–family conflict literature and answers Amstad et al.’ s [96] call to strengthen studies on the antecedent variables of work–family conflict, thus providing a new perspective for understanding the work–family conflict of Chinese expatriates in cross-cultural NPOs.

Third, based on COR theory, this study found that LMX serves to buffer the positive relationship between work engagement and work–family conflict for expatriates in a cross-cultural NPO. Many studies have shown that work–family balance mainly depends on the intensity of the physical and psychological boundaries that individuals encounter every day on moving between the work and family domains [97,98]. However, few studies have explored the specific variables affecting the strength of these boundaries, and LMX as a boundary condition for individuals balancing work–family relationships has never been explored. This study confirms that LMX can affect the strength of the boundary between the work and family domains, which not only fills a research gap but also defines a boundary condition for understanding how cultural intelligence impacts on work–family conflict in cross-cultural NPOs. In other words, this study theoretically demonstrates under what conditions cultural intelligence is more likely to exert an influence on expatriates’ work–family conflict. This conclusion echoes Wayne et al.’s [99] findings that emphasize leaders’ importance for employees’ work–family conflict, and also answers their call for studies to further explore the moderating variables between work engagement and negative outcomes [2,33]. The results of the moderated mediation model also deepen understanding of the complex role of cultural intelligence [24]. Theoretically, this study provides a new perspective for understanding the boundary conditions between work engagement and work–family conflict. It also provides important insights for organizations to solve the dilemma of employees’ potential work–family conflict while promoting their work engagement.

### 5.2. Practical Implications

This study yields important implications for HRM in cross-cultural NPOs, specifically the management of work–family conflict among expatriates in such organizations. First, this study found that cultural intelligence can promote employees’ work engagement and reduce their work–family conflict. As a malleable skill, cultural intelligence has always been seen as a valuable asset in the cross-cultural workplace. Therefore, considering the importance of cultural intelligence, managers or organizations should focus on cultural intelligence promotion and training among employees. At the organizational level, cross-cultural organizations should consider building a strong organizational culture across all levels of management, especially emphasizing values of flexibility, diversity, tolerance, and cooperation, so as to help employees develop and apply the full range of their cultural intelligence repertoires. These measures can be reinforced by establishing policies or practices that encourage employee to openly communicate and to learn from other cultures [100]. Considering the importance of pre-departure training in shaping cultural skills [101], organizations could provide pre-departure cultural intelligence training for expatriates, thereby helping to reduce potential culture shocks and interpersonal conflicts. Such training should include multicultural elements, provide more opportunities for cross-cultural cooperation between employees or teams, and increase employees’ involvement in cross-cultural social interaction. This would help to promote expatriates’ cross-cultural adaptation and enhance their cultural intelligence.

After expatriates enter the overseas organization, managers should provide more opportunities for them to enhance and apply their cultural intelligence through various formal or informal intercultural activities (e.g., team building). Managers should assist expatriates to understand and learn the characteristics of other cultures; stimulate their interest in interacting with colleagues, customers, or suppliers from different cultural backgrounds; and guide them on how to communicate properly across cultures. Further, managers should include cultural intelligence as an important criterion for evaluating expatriates’ performance and establish corresponding incentive policies. Employees with excellent cultural intelligence performance should be encouraged or rewarded by organizations. Through these efforts, we believe that managers could gradually help to improve expatriates’ cross-cultural competence (cultural intelligence).

Second, as work engagement was found to play a mediating role in the cultural intelligence and work–family conflict relationship, employees with high work engagement likely face more challenges in balancing work and family life, which LMX can alleviate. Therefore, organizations should especially seek to establish and develop high-quality LMX between supervisors and subordinates while encouraging employees’ work engagement. On the one hand, managers should recognize the two reversible relationships between expatriates’ cultural intelligence and work engagement, and attach importance to promoting employees’ work engagement through cultural intelligence training projects. On the other hand, organizations should develop high-quality LMX by providing opportunities for enhancing the interaction between leaders and subordinates. For example, leaders should be encouraged to participate in various activities or events for subordinates, such as festival gatherings or birthday parties hosted by the organization. Additionally, managers should provide flexible and supportive policies to help employees with work–family balance and create a family-supportive organizational climate for employees [94]. For example, leaders could develop high LMX with subordinates by providing flexible working hours, especially on some important holidays.

### 5.3. Limitations and Future Research Directions

This study is not without limitations. First, although it employs a longitudinal design with time-lagged data to test the hypotheses, the two-waves assume a linear relationship between the study variables. This limits our conceptualization of change to a linear function [102] (p. 377). Future studies could use other time intervals for measuring the study variables to further understand how cultural intelligence influences work–family conflict.

Second, data in this research were self-reported and single-sourced, which raises CMV concerns. Although the results of CMV tests are well within acceptable thresholds, diverse data sources are more desirable in work–family research [103]. Future studies could adopt a multi-source evaluation method to measure variables: For example, leader-rated LMX is usually considered more rigorous than subordinate-rated LMX in research design [104].

Third, the global distribution and regional differences of Confucius Institutes raise concerns regarding the study’s external validity. Because the relatively small sample (*N* = 206) only includes expatriate Chinese teachers working at Confucius Institutes in the US, Canada, and Russia, future studies are needed to verify whether our theoretical model and findings are applicable to other groups, such as expatriates in other Confucius Institutes or those from other cultures or in alternative enterprises. Future studies could conduct cross-cultural validation of our findings by expanding the sample population or increasing the diversity of its composition.

Fourth, other variables or underlying theories that might explain the influence mechanism of cultural intelligence on work–family conflict require further investigation. For example, considering the impact of gender on work–family conflict, role theory would be a useful perspective to adopt in exploring work–family conflict [105]. Finally, the impact of cultural intelligence on work–family conflict may be affected by individual differences. Besides LMX, future studies could focus more on individual difference as boundary conditions, such as traditionality or social roles [24]. In short, these are fruitful avenues of future research that warrant exploration.

## 6. Conclusions

This study contributes to HRM research by examining the influence mechanism of cultural intelligence on work–family conflict for expatriates in a cross-cultural NPO. The results suggest that cultural intelligence not only promotes expatriates’ work engagement but also reduces their work–family conflict, thereby further highlighting cultural intelligence’s importance for expatriates. Organizations and HR managers are advised to introduce effective training programs and incentive policies to promote employees’ cultural intelligence. Future studies are needed to verify our findings’ application to expatriates from various other cultures. This study has important implication for managers seeking to understand and solve the work–family conflict problem faced by many expatriates. We believe that exploring additional antecedents of work–family conflict is a promising avenue for future studies.

## Figures and Tables

**Table 1:** Descriptive statistics and correlations among study variables.

Variables	Mean	SD	1	2	3	4	5	6	7	8	9
1. Gender	0.31	0.462	1								
2. Age	2.95	1.818	0.064	1							
3. Education level	2.25	0.563	−0.111	0.051 *	1						
4. Tenure	3.16	1.302	0.072	−0.012	0.002 *	1					
5. Country	0.797	0.46	0.032	0.041	0.013	0.067	1				
6. CQ	3.546	0.982	−0.12	−0.006	0.208 *	0.067	0.078	(0.91)			
7. WFC	3.213	0.97	−0.07 **	0.03 **	0.114 **	−0.025 **	0.019	−0.450 **	(0.83)		
8. WE	3.122	0.767	−0.12	−0.014	0.200 *	0.058 **	0.072 *	0.337 **	0.295 **	(0.85)	
9. LMX	3.658	0.598	−0.107	0.011	0.208	0.11	0.092	0.225 **	−0.298 *	0.312 **	(0.94)

*Note. N* = 206; CQ = cultural intelligence, WFC = work–family conflict, WE = work engagement, LMX = leader–member exchange. Gender is coded as 0 = female, 1 = male. Age is coded as 1 = 18–25 years, 2 = 26–30 years, 3 = 31–35 years, 4 = 36–40 years, 5 = 41–45 years, 6 = 46 years and above. Education level is coded as 1 = bachelor’s degree, 2 = master’s student/graduate, 3 = Ph.D. student/graduate. Tenure is coded as 1 = 0–3 months, 2 = 3 months–1 year, 3 = 1–2 year(s), 4 = 2–4 years, 5 = 5 years and above. Country is coded as 1 = America, 2 = Canada, 3 = Russia. * *p* < 0.05, ** *p* < 0.01.

**Table 2:** Results of confirmatory factor analysis.

Measurement Models	*x* ^2^	*df*	*x*^2^/*df*	CFI	TLI	IFI	RMSEA
Baseline model	876.528	318	2.756	0.914	0.905	0.914	0.078
Unmeasured latent factor model	728.358	298	2.444	0.921	0.909	0.922	0.072
Three-factor model	1479.501	321	4.609	0.821	0.804	0.822	0.112
Two-factor model	2030.720	323	6.287	0.736	0.714	0.738	0.136
One factor model	2613.072	324	8.065	0.647	0.617	0.648	0.157

*Note.* The baseline model includes the study’s four variables (cultural intelligence, work-engagement, work–family conflict, and LMX); the unmeasured latent factor model includes another common variance factor based on the baseline model; the three-factor model is cultural intelligence + work-engagement, work–family conflict, LMX; the two-factor model is cultural intelligence + work-engagement, work–family conflict + LMX; the one factor model is cultural intelligence + work-engagement + work–family conflict + LMX, “+” indicates that variables are combined into a single model.

**Table 3:** Results of multiple regression analysis.

Variables	Work–Family Conflict						Work Engagement	
	Model 1	Model 2	Model 3	Model 4	Model 5	Model 6	Model 7	Model 8
Control								
Gender	−0.058 *	0.01	0.015 **	0.013 *	0.014	0.027	−0.103 *	−0.002
Age	0.028	0.036	0.041	0.045 *	0.059 **	0.066 **	−0.018	−0.007
Education level	0.107	−0.024	−0.027 *	−0.02	−0.006 *	−0.008 *	0.19	−0.003
Tenure	−0.021 *	−0.071	−0.067	−0.061	−0.022	−0.019	0.066 *	−0.008
Country	0.024	0.021	0.023	0.018	0.019	0.022	−0.068	−0.034
Independent variable								
CQ		−0.560 ***		−0.434 *	−0.040	−0.052		0.488 ***
Mediator and moderator								
WE			0.506 ***	0.423 ***	0.572 ***	0.541 ***		
LMX					−0.414 ***	−0.308 ***		
WE x LMX						−0.152 **		
*R²*	0.022	0.410	0.472	0.486	0.552	0.567	0.056	0.392
*△R^2^*	0.022	0.388	0.450	0.076	0.530	0.015	0.056	0.336
F	0.883 *	25.050 ***	32.073 ***	28.965 ***	36.710 ***	34.574 ***	3.020 *	54.737 ***
*△*F	0.883 *	142.758 ***	183.985 ***	20.312 ***	94.361 ***	7.619 **	3.020 *	307.318 ***

*Note. N* = 206. Standardized coefficients are reported. CQ = cultural intelligence, WE = work engagement, LMX = leader–member exchange. *** *p* < 0.001, ** *p* < 0.01, * *p* < 0.05 (two-tailed).

**Table 4:** Results of the moderated path analysis.

Moderator	CQ (X)→WE (M)→WFC (Y)				
	Stage		Effect		
	First	Second	Direct	Indirect	Total
	PMX	PYM	PYX	PYM × PMX	PYX + PYM × PMX
Low-level LMX (−1SD)	0.622 **	0.591 **	0.352 **	0.367 **	0.719 **
High-level LMX (+1SD)	0.541 **	0.287 **	0.366 **	0.156 **	0.522 **
Difference	−0.081	−0.304 *	0.014	−0.211 *	−0.197 **

*Note. N* = 206. LMX = leader–member exchange, CQ = cultural intelligence, WE = work engagement, WFC = work–family conflict; “→”refers to the path of effect from one variable to another; PMX: Path from cultural intelligence to work engagement; PYM: Path from work engagement to work–family conflict; PYX: Path from cultural intelligence to work–family conflict. Low-level LMX refers to one standard deviation below the LMX mean; high-level LMX refers to one standard deviation above the LMX mean. ** *p* < 0.01, * *p* < 0.05 (two-tailed).
